# Cytomegalovirus Seropositivity Is Associated with Increased Arterial Stiffness in Patients with Chronic Kidney Disease

**DOI:** 10.1371/journal.pone.0055686

**Published:** 2013-02-25

**Authors:** Nadezhda A. Wall, Colin D. Chue, Nicola C. Edwards, Tanya Pankhurst, Lorraine Harper, Richard P. Steeds, Sarah Lauder, Jonathan N. Townend, Paul Moss, Charles J. Ferro

**Affiliations:** 1 School of Immunity and Infection, University of Birmingham, Birmingham, United Kingdom; 2 Department of Nephrology, Queen Elizabeth Hospital Birmingham, Birmingham, United Kingdom; 3 School of Clinical and Experimental Medicine, University of Birmingham, Birmingham, United Kingdom; 4 Department of Cardiology, Queen Elizabeth Hospital Birmingham, Birmingham, United Kingdom; 5 School of Cancer Sciences, University of Birmingham, Edgbaston, Birmingham, United Kingdom; University of Regensburg, Germany

## Abstract

**Background:**

Patients with chronic kidney disease have an increased cardiovascular risk that is not fully explained by traditional risk factors but appears to be related to increased arterial stiffness. Cytomegalovirus (CMV) infection is associated with increased cardiovascular risk although the mechanisms for this are unknown. We examined whether CMV seropositivity was associated with increased arterial stiffness in patients with chronic kidney disease.

**Methodology and Principal Findings:**

In 215 non-diabetic patients with chronic kidney disease, CMV seropositivity was determined using an anti-CMV IgG ELISA. Pulse wave velocity was measured and aortic distensibility assessed in the ascending, proximal descending and distal descending thoracic aorta. Patients seropositive for CMV had a higher pulse wave velocity and lower aortic distensibility at all 3 levels. These differences (except for ascending aortic distensibility) persisted in a subcohort matched for age, gender and renal function, and when the whole cohort was divided into quartiles of age. In multivariable analyses, CMV seropositivity was an independent determinant of pulse wave velocity and proximal and distal descending aortic distensibility.

**Conclusions:**

In patients with chronic kidney disease, CMV seropositivity is associated with increased arterial stiffness and decreased distensibility of the proximal descending and distal aorta. These findings suggest that further research is required to examine CMV as a possible cause of arterial disease and increased cardiovascular risk in patients with CKD and may be relevant more widely for CMV seropositive patients with normal renal function.

## Introduction

Chronic kidney disease (CKD) is a global public health problem affecting over 13% of the western population [1]. Cardiovascular (CV) risk is increased in CKD with numerous observational studies demonstrating an independent, graded inverse correlation between estimated glomerular filtration rate (eGFR) and increasing CV event rates [2]. This relationship is not fully explained by traditional cardiovascular risk factors [3]. The phenotype of CV disease associated with CKD is multi-factorial. Premature atherosclerosis causing vascular occlusive events is prevalent in this condition [4]. In contrast to atheroma, which affects the vascular intima, arteriosclerosis is a disease of the arterial medial layer in which increased collagen content, together with calcification, hyperplasia and hypertrophy of vascular smooth muscle cells (VSMC), lead to arterial wall hypertrophy and increased arterial stiffness [3]. The severity of arteriosclerosis, assessed using indices of arterial stiffness, is increased in patients with CKD and is a powerful marker of mortality in this condition [3], [5].

Cytomegalovirus (CMV) is a member of the herpes virus family and is widely prevalent in the human population. Seropositivity rates increase with both age and socioeconomic deprivation [6]. Following initial infection the virus is not eradicated from the host and establishes a state of chronic infection with episodes of intermittent reactivation. The potential role of CMV infection as a risk factor for cardiovascular disease (CVD) is controversial. Significant associations have been reported between CMV seropositivity and CVD risk [7]–[9], with the strongest associations observed in patients undergoing immunosuppressive treatment following organ transplantation [10], [11], However, not all studies have demonstrated this association [12], [13].

Three studies have shown an association between CMV seropositivity and carotid artery distensibility in univariate analysis [14]–[16]. However, the relationship only remained positive after multivariate adjustment in HIV positive, but not negative, women [16]. To the best of our knowledge, any potential association between CMV seropositivity and aortic stiffness has never been examined. We therefore explored this relationship in a cohort of CKD patients using carotid-femoral pulse wave velocity (PWV), the current gold-standard measure of arterial stiffness [5], and aortic distensibility at three different levels as a second measure.

## Methods

### Study design, setting and participants

Patients were prospectively recruited from renal clinics at the Queen Elizabeth Hospital Birmingham, UK. Patients were included if aged 18–80 years with CKD stages 2–4 (eGFR 15–89 ml/min/1.73 m^2^ estimated using the four-variable Modification of Diet in Renal Disease formula). Patients with a history or other evidence of angina, previous myocardial infarction, previous stroke, peripheral vascular disease, previous revascularisation procedure, heart failure, atrial fibrillation, moderate or severe cardiac valve disease, uncontrolled hypertension (mean daytime 24-hour ambulatory blood pressure (BP) >130/85 mmHg), hypercholesterolemia (total serum cholesterol >5.5 mmol/L) and diabetes mellitus were excluded. The West Midlands Research Ethics Committee approved the study and written informed consent was obtained from each participant.

### Blood pressure measurements

Brachial BP was recorded in the non-dominant arm in triplicate following 15 minutes of supine rest using a validated oscillometric sphygmomanometer (Dinamap ProCare 200, GE Healthcare, United Kingdom). All subjects underwent 24-hour ambulatory BP measurement (Meditech ABPM-04; PMS Instruments, Maidenhead, UK).

### Pulse wave analysis and pulse wave velocity

Central pressure waveforms were derived and analysed using the technique of pulse wave analysis (SphygmoCor, Atcor Medical, Sydney, Australia) as previously described [17]. Aortic PWV was measured using the SphygmoCor system by sequentially recording ECG-gated carotid and femoral artery waveforms. The path length was calculated by subtracting the distance between the sternal notch and carotid recording site from the distance between sternal notch and femoral site.

### Aortic distensibility

Aortic distensibility was measured using cardiovascular magnetic resonance imaging (CMR) at 1.5 Tesla (Symphony, Siemens, Erlangen, Germany). Steady-state free precession, R-wave gated, sagittal-oblique cine sequences were undertaken with the following parameters: temporal resolution 50–60 ms, echo time 2.2 ms, flip angle 60°, field of view 300 mm and slice thickness of 5 mm. Analysis was performed offline (Argus Software, Siemens, Erlangen, Germany) by two observers blinded to CMV status. Area measurements were performed in triplicate at the ascending and proximal descending thoracic aorta at the level of the pulmonary artery and at the distal descending thoracic aorta at the diaphragm. Aortic distensibility (×10^−3^ mmHg^−1^) was calculated using the standard formula [18]:
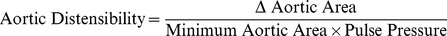
where Δ Aortic Area = (Maximum Aortic Area−Minimum Aortic Area) and Pulse Pressure is the average of three brachial pulse pressure measurements performed synchronously with image acquisition using a non-ferromagnetic cuff in the non-dominant arm.

### Cytomegalovirus assay

The CMV IgG status of patients was evaluated using an in-house enzyme-linked immunosorbent assay. A 96 well plate was coated with cell lysate purified from CMV-infected fibroblast cultures, with lysate from uninfected cells used as a negative control (50 µL/well diluted in coating buffer (0.2M sodium carbonate/0.2M sodium bicarbonate/pH 9.6) and incubated at 4°C for 16 hours). Samples were added in a 1∶600 dilution in buffer (phosphate buffered saline (PBS)/1% bovine serum albumin/0.05% Tween20) together with standards to make a calibration curve (pooled plasma from three healthy CMV positive donors). Secondary antibody (anti-human IgG-horseradish peroxidase, Southern Biotech) was added after 30 minutes incubation at room temperature and washing with PBS/0.05% Tween20. Tetramethylbenzidine solution was added after a further 30 minutes/wash and incubated for 10 minutes at room temperature, protected from light. The reaction was stopped using 1M hydrochloric acid and the plate was read immediately using a microplate reader at absorbance 450 nm. Optical density was analysed using GraphPad PRISM (GraphPad Software, CA) and values attributable only to CMV IgG were calculated by subtracting control lysate values from that of the CMV lysates. A cut off of 10 arbitrary units was used to determine positive/negative CMV IgG status.

### Statistical analyses

Baseline characteristics were assessed using standard descriptive statistics. Data distribution was tested using the Kolmogorov-Smirnov test. Data are presented as mean±standard deviation or median (interquartile range) for normally or non-normally distributed variables respectively. Variables not normally distributed were log transformed prior to analysis. Colinearity between variables was assessed by examining the variance inflation factor; a value >5 indicated colinearity. Linear regression was used to examine the relationships between measures of arterial stiffness and baseline demographic parameters. We utilized multivariable regression models to examine the relationship between arterial stiffness parameters and significant correlates on univariable analysis. A Type I error rate below 5% (*P*<0.05) was considered statistically significant. All data were analysed using SPSS version 20 (SPSS Inc, Chicago, IL).

Studying 98 patients in each group provided 95% power to detect a difference in PWV of 0.4 m/s between groups based on our previously published work on arterial stiffness in CKD [17], where mean PWV was 8.3±1.7 m/s using a two-tailed t-test at the 5% significance level.

## Results

### Patient characteristics

A total of 215 patients were recruited; mean age was 55±13 years with 59% male and 88% Caucasian, with 12% being South Asian. Excluding non Caucasian patients made no appreciable difference to any of the subsequent analyses and therefore results for the whole cohort are presented. Table 1 depicts demographic and laboratory data of all subjects and according to CMV seropositivity. Thirty-two (15%) subjects were current smokers with 64 (30%) being ex-smokers.

**Table 1:** Patient demographics, hematological and biochemical variables according to CMV seropositivity.

	CMV positive n = 119	CMV negative n = 96	P
Male gender (%)	68 (58)	56 (58)	1.0
Age (years)	57±13	51±12	<0.001
eGFR (ml/min/1.73 m^2^)	50±16	51±16	0.8
hsCRP (µg/mL)*	2.1 (1.0–6.6)	1.9 (0.5–5.5)	0.6
Peripheral SBP (mmHg)	131±18	127±16	0.1
Peripheral DBP (mmHg)	75±11	73±11	0.5
Central SBP (mmHg)	122±18	116±17	0.02
Central DBP (mmHg)	75±11	74±11	0.5
24 hour SBP (mmHg)	123±12	123±12	1.0
24 hour DBP (mmHg)	72±9	73±8	0.3
Heart rate (bpm)	65±12	64±11	0.4
AIx (%)	30±11	25±11	0.003
AIx_75_ (%)	25±10	20±12	0.001
Pulse wave velocity (m/s)	9.7±3.1	8.2±2.0	<0.001
Ascending AoD (×10^−3^ mmHg^−1^)	2.23±1.87	3.11±1.75	0.003
Proximal descending AoD (×10^−3^ mmHg^−1^)	2.84±1.42	3.83±1.64	<0.001
Distal descending AoD (×10^−3^ mmHg^−1^)	3.71±1.70	4.98±2.32	<0.001

* Log transformed prior to analysis. Data are frequency (percentage), mean±standard deviation or median (interquartile range). Data analysed using unpaired two-tailed Student's t-test or Pearson's χ^2^. CMV, cytomegalovirus; eGFR, estimated glomerular filtration rate; hsCRP, high sensitive C-reactive protein; SBP, systolic blood pressure; DBP, diastolic blood pressure; AIx, augmentation index; AIx_75_, augmentation index adjusted to heart rate of 75 bpm; AoD, aortic distensibility.

Seropositivity for CMV IgG antibody was present in 119 patients (55%) (Table 1). No significant differences were observed between seronegative and seropositive patients in relation to gender, ethnic origin, smoking or alcohol status. CMV seropositive patients were older (57±13 years) than seronegative patients (51±12 years; P<0.001). CMV positive and negative patients were taking the same number of antihypertensive agents (1.8±1.1 v. 1.8±1.2 respectively; P = 1.0). One hundred and ninetyeight (92%) patients were taking at least one antihypertensive agent with 55% taking 2 or more antihypertensive agents. There were no differences in the proportion of patients taking different types of antihypertensive medication (angiotensin converting enzyme inhibitor, angiotensin receptor blockers, beta-blockers, alpha-blockers or diuretics; P>0.2 for all).

Biochemical variables are summarized in Table 1. There were no differences in eGFR, serum glucose, total cholesterol, low-density lipoprotein (LDL), high-density lipoprotein (HDL), HDL/LDL ratio serum calcium, phosphate, parathyroid hormone, high sensitive C-reactive protein (hsCRP), urinary albumin: creatinine ratio and haemoglobin between CMV seropositive and seronegative patients.

### Hemodynamics

The hemodynamic parameters for both groups are summarized in Table 1. Office brachial and 24-hour BP were not different between CMV seropositive and seronegative patients. Central aortic systolic BP and pulse pressure were, however, higher in seropositive patients. Patients with CMV seropositivity had higher augmentation index (AIx), AIx_75_ (AIx adjusted to a heart rate of 75 bpm) and PWV. Aortic distensibility was also significantly lower in the ascending, proximal descending and distal descending aorta in seropositive patients compared with seronegative patients. Office and 24-hour heart rates were not different between groups.

### Cytomegalovirus status and arterial stiffness in age quartiles

**Figure 1:**
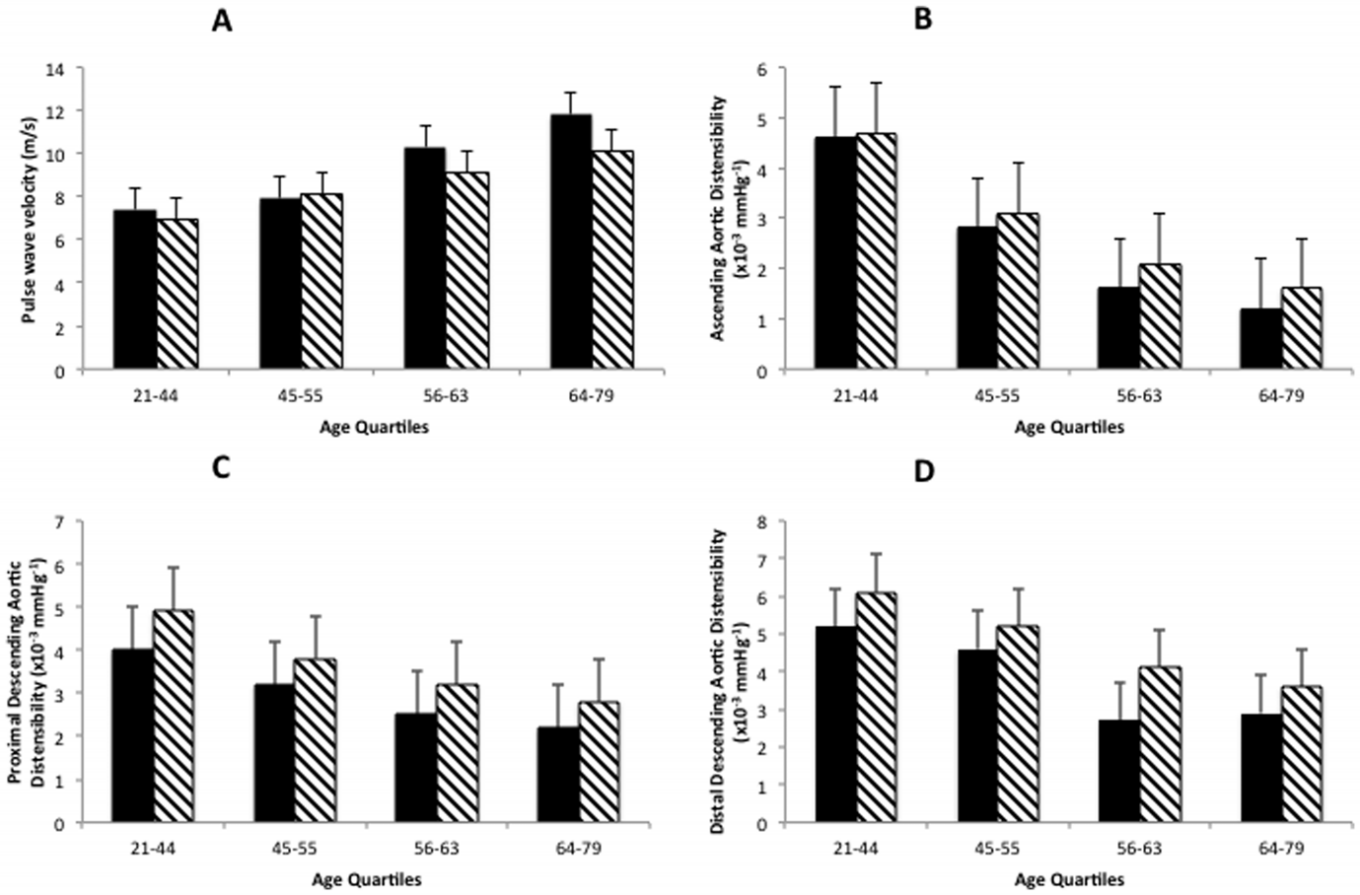
Arterial stiffness across age quartiles in CMV positive (black columns) and CMV negative patients (hashed columns). (A) Pulse wave velocity increases with age (P<0.001) and is higher in CMV positive patients (P = 0.02). (B) Ascending aortic distensibility decreases with age (P<0.001) but is not significantly lower in CMV seropositive patients (P = 0.1). (C and D) Proximal and distal descending aortic distensibility decrease with age (P<0.001) and are significantly lower in CMV positive patients (P<0.001).

To account for differences in age between CMV seropositive and seronegative groups, the cohort was divided into quartiles according to age and analyzed by two-way analysis of variance (Figure 1). PWV velocity increased with age (P<0.001) and was consistently higher in CMV positive patients (P = 0.02; Figure 1A). Cytomegalovirus seropositivity increased PWV by an average of 0.7 (0.1–1.4) m/s. Ascending aortic distensibility decreased with age (P<0.001) but was not significantly different in CMV patients (P = 0.1; Figure 1B). Aortic distensibility in the proximal and distal descending aorta (Figures 1C and 1D respectively) decreased with age and CMV seropositivity (P<0.001 for both). There were no significant interactions between CMV seropositivity and age on PWV or aortic distensibility at the 3 levels. There were no differences in 24 hour ambulatory blood pressure, brachial blood pressure or central blood pressure across the quartiles.

### Impact of CMV seropositivity in an age-matched cohort of CKD patients

Because age could have confounded our results we carefully matched 120 patients for gender, age (within 2 years) and eGFR (within 5 ml/min/1.73 m^2^) [19]. Clinical characteristics and hemodynamic parameters are presented in Table 2. Despite similar BP, CMV seropositive patients had higher AIx, AIx_75_ and PWV. Both proximal and distal descending aortic distensibility were reduced in CMV positive patients (P = 0.01 for both).

**Table 2:** Patient demographics for 60 patient pairs matched for gender and age.

	CMV positive n = 60	CMV negative n = 60	P
Male (%)	26 (43)	26 (43)	1.0
Age (years)	55±9	55±9	1.0
eGFR (ml/min/1.73 m^2^)	50±17	50±16	1.0
hsCRP (µg/mL)*	2.68 (1.01–6.62)	1.39 (0.50–4.52)	0.2
Brachial SBP (mmHg)	132±20	127±1	0.1
Brachial DBP (mmHg)	76±10	75±9	0.4
Central SBP (mmHg)	124±20	118±15	0.07
Central DBP (mmHg)	77±10	76±9	0.4
24-hour SBP (mmHg)	124±12	122±11	0.2
24-hour DBP (mmHg)	74±9	73±8	0.6
AIx (%)	31±12	27±9	0.04
AIx_75_ (%)	26±10	22±9	0.02
PWV (m/s)	9.2±2.1	8.2±1.3	0.03
Ascending AoD (×10^−3^ mmHg^−1^)	2.24±1.59	2.66±1.56	0.2
Proximal descending AoD (×10^−3^ mmHg^−1^)	2.83±1.34	3.52±1.44	0.01
Distal descending AoD (×10^−3^ mmHg^−1^)	3.83±1.85	4.86±2.32	0.01

* log transformed before analysis. CMV, cytomegalovirus; eGFR, estimated glomerular filtration rate; hsCRP, high sensitive C-reactive protein; SBP, systolic blood pressure; DBP, diastolic blood pressure; AIx, augmentation index; AIx_75_, augmentation index adjusted to heart rate of 75 bpm; PWV, pulse wave velocity; AoD, aortic distensibility.

### Cytomegalovirus status as a determinant of arterial stiffness

In univariate analysis, PWV was strongly associated with CMV positive status (B = 1.44, 95% confidence interval (CI) 0.3–2.18, P<0.001). Pulse wave velocity was also associated with brachial, central and 24-hour systolic BP, mean arterial and pulse pressures, age, eGFR, HDL cholesterol, parathyroid hormone, albumin: creatinine ratio and hsCRP. These parameters were entered into a stepwise regression analysis. As expected, all BP measures exhibited significant colinearity, therefore only one parameter was entered into the model at a time. Central pulse pressure was entered into the presented model as the most highly correlated BP parameter. In multivariate analysis (Table 3) PWV remained positively associated with central pulse pressure, age and CMV status (B = 0.67, 95% CI 0.04–1.21, P = 0.03). Substituting central systolic, brachial or 24-hour systolic BP or pulse pressures made no appreciable difference to the analyses.

**Table 3:** Multiple stepwise regression analysis for (A) pulse wave velocity, (B) ascending aortic distensibility, (C) proximal descending aortic distensibility and (D) distal descending aortic distensibility.

	Unstandardised coefficients		Standardised coefficients	T	P
	B	SE	β		
(A) Pulse wave velocity (adjusted R^2^ for model 0.49)					
Age (years)	0.01	0.01	0.49	7.64	<0.001
Central PP (mmHg)	0.05	0.01	0.29	4.54	<0.001
CMV seropositivity	0.67	0.31	0.13	2.19	0.03
Independent variables: age, central PP, CMV seropositivity (yes = 1), eGFR, log PTH, log ACR, log hsCRP					
(B) Ascending aortic distensibility (adjusted R^2^ for model 0.54)					
Age (years)	−0.09	0.01	−0.61	−7.94	<0.001
Central PP (mmHg)	−0.03	0.01	−0.20	−2.63	0.01
Gender	0.59	0.28	0.15	2.16	0.03
Independent variables: age, central PP, CMV seropositivity (yes = 1), gender (male = 1), serum calcium					
(C) Proximal descending aortic distensibility (adjusted R^2^ for model 0.33)					
Age (years)	−0.05	0.01	−0.39	−5.64	<0.001
Central PP (mmHg)	−0.02	0.01	−0.22	−3.27	0.001
CMV seropositivity	−0.55	0.02	−0.17	−2.73	0.007
Independent variables: age, central PP, CMV seropositivity (yes = 1), log hsCRP					
(D) Distal descending aortic distensibility (adjusted R^2^ for model 0.31)					
Age (years)	−0.05	0.01	−0.33	−4.80	<0.001
Central PP (mmHg)	−0.04	0.01	−0.27	−3.92	<0.001
CMV seropositivity	−0.74	0.27	−0.18	−2.75	0.007
Independent variables: age, central PP, CMV seropositivity (yes = 1), log hsCRP					

B, unstandardized co-efficient; SE, standard error; β, standardized co-efficient; PP, pulse pressure; CMV, cytomegalovirus; GFR, glomerular filtration rate; PTH, parathyroid hormone; ACR, urinary albumin: creatinine ratio; hsCRP, high sensitive C-reactive protein.

Cytomegalovirus seropositivity was inversely associated with ascending (B = −0.82, 95% CI −1.35–−0.29, P = 0.003), proximal descending (B = −0.99, 95% CI −1.43–−0.55, P<0.001) and distal descending (B = −1.27, 95% CI −1.85–−0.68, P<0.001) aortic distensibility in univariate analyses. In multivariate analysis ascending aortic distensibility was not significantly associated with CMV seropositivity. Both proximal (B = −0.55, 95% CI −0.9–−0.15, P = 0.007) and distal descending aortic distensibility (B = −0.74, 95% CI −1.27–−0.21, P = 0.007) remained associated with CMV positivity after multivariate adjustment. Central pulse pressure was used in these models because it had the strongest univariate correlation with aortic distensibility. Substituting central systolic, brachial or 24-hour systolic BP or pulse pressures made no appreciable difference to the analyses.

## Discussion

In patients with CKD, seropositivity for CMV was positively associated with PWV, the gold-standard measure of arterial stiffness. Furthermore, CMV seropositivity was consistently associated with decreased distensibility of the proximal and distal descending aorta, but not the ascending aorta. The increased arterial stiffness associated with CMV seropositivity together with the differential effects on aortic segments could provide novel insights into the pathophysiology of increased arterial stiffness in CKD and potentially in various disease states.

The powerful prognostic significance of increased arterial stiffness is well recognized [3], [5], Failure to buffer adequately intermittent left ventricular ejection into the arterial system results in left ventricular hypertrophy and fibrosis, cerebrovascular disease and further renal damage [3], [5]. Many potential mechanisms have been postulated to contribute to the increased arterial stiffness associated with CKD [3]. Our results suggest that past infection with CMV may be a potentially modifiable CV risk factor.

The effects of CMV on arterial wall function might be mediated via actions within the arterial media, either by changing VSMC properties or by causing inflammation and fibrosis. Histopathological studies have reported evidence of CMV particles in the whole human vascular tree in CMV seropositive patients [20]–[24]. Vascular smooth muscle cells can be infected by CMV leading to a viral arteritis [21], [22]. Alternatively, previous CMV infection may alter the function of vascular smooth muscle cells permanently. Relatively little is known about the cell tropism of CMV *in vivo* but the endothelium is certainly a reservoir for infection. Indeed, CMV-specific T cells demonstrate the characteristic feature of CX3CR1 expression that targets these cells to stressed endothelial cells through fractalkine binding [25]. Some studies [26], [27], although not all [28], [29], have also shown an association between CMV seropositivity and endothelial dysfunction. We did not include any measures of endothelial function in our study but this is certainly an area that warrants further investigation.

Chronic inflammation has long been recognized as a cardiovascular risk factor and a clear association also exists between inflammation and arterial stiffness, as demonstrated by studies of conditions characterized by chronic systemic inflammation including CKD and in the general population [3]. More specifically, aortic inflammation, as assessed using positron emission tomography imaging, has recently been shown to influence aortic PWV [30]. Although only measured at a single time point, we found no differences in hsCRP or serum albumin concentrations between CMV seropositive and seronegative patients in our analyses. This does not support chronic inflammation as a possible explanation for our findings. Chronic kidney disease is associated with the relative accumulation of many serum proteins and it is possible that even mild states of CKD could be associated with increased rates of sub-clinical CMV reactivation, although this has not been investigated in this patient group [31].

A very close relationship exists between arterial stiffness and BP and this raises the possibility of whether or not CMV infection may also directly influence BP through secondary effects on arterial wall function. Interestingly, high CMV antibody titres have recently been shown to be independently associated with increased BP in healthy young Finnish men but not women [32]. Furthermore, CMV ribonucleic acid copy number was associated with hypertension in a Chinese cohort [33]. Nevertheless, our finding of an increase in arterial stiffness associated with CMV seropositivity was independent of BP, suggesting a direct effect on blood vessels themselves. Interestingly, a murine model of CMV infection is associated with increased blood pressure independent of a high cholesterol diet and atheroma formation. In addition to stimulating expression of inflammatory cytokines, CMV infection also increased the synthesis of renin and angiotensin II [34]. The renin-angiotensin-aldosterone system is known to increase arterial stiffness and this is an area that warrants further investigation [3].

The visco-elastic properties of the aorta vary along its length, with a gradual decrease in both collagen and elastin content from proximal to distal [3]. Furthermore, it is becoming increasingly recognized that VSMC in different arteries, or indeed portions of the same artery, have different phenotypic properties and embryonic origins. Vascular smooth muscle cells in the ascending aorta and arch derive from neural crest, whereas those in the descending aorta have a somitic origin [35]. In addition, VSMC from different embryonic origins respond in lineage-specific ways to common stimuli [35] and may well vary in both their relative tropism for, and metabolic response to, CMV infection. Importantly, VSMC phenotype has already been shown to directly determine susceptibility to CMV infection [36]. Our finding of an apparent different association between CMV seropositivity and distensibility at different aortic levels warrants confirmation in larger studies.

Our study has a number of limitations. Most subjects were taking antihypertensive medication, as would be expected, and this could potentially affect the biophysical properties of the aorta. Despite this, however, we were still able to detect significant differences in PWV and aortic distensibility between CMV seropositive and seronegative patients. Our study was not powered to examine the influence of individual antihypertensive agents on arterial stiffness. We used peripheral brachial pulse pressures in the calculations of aortic distensibility as it was not technically possible to obtain central aortic pressures during image acquisition. Aortic distensibility values are slightly lower when brachial pulse pressures are used [37] although we do not believe this would have appreciably affected the overall significance of our results. Given the strong relationship with socio-economic status and CMV exposure, consideration of socio-economic status in relation to vascular function would have been a valuable measurement [6]. It is possible that measuring CMV DNA might have given some more information on active CMV infection/reactivation. However, examining the potential effects of viral load would require a much larger study. Similarly, the CMV assay we used does not quantify CMV antibody titres which could also have yielded potentially interesting information. Our study was performed in patients with CKD who are known to have increased arterial stiffness. Our results therefore also need validating in other populations. Finally, our study is cross-sectional in design and therefore only significant associations and not causality can be determined.

In summary, we have shown that CMV seropositivity is associated with increased arterial stiffness in a cohort of patients with early stage CKD, independent of age and blood pressure. Using a complementary imaging modality, we also observed a reduction in distensibility of the proximal and distal descending aorta. These findings have significant potential implications for the mechanism by which CMV infection might influence cardiovascular disease. Although confirmation in larger cohorts is required, our results highlight the fact that CMV seropositivity may not be as trivial as is currently considered in non-heavily immunosuppressed individuals. Ultimately, reducing the prevalence of CMV seropositivity might be a potential way of reducing the burden of cardiovascular disease in the general population.
